# Scalable in-memory processing of omics workflows

**DOI:** 10.1016/j.csbj.2022.04.014

**Published:** 2022-04-20

**Authors:** Vadim Elisseev, Laura-Jayne Gardiner, Ritesh Krishna

**Affiliations:** a IBM Research Europe, Hartree Centre, Daresbury Laboratory, Keckwick Lane, WarringtonWA4 4AD, Cheshire, UK; b Wrexham Glyndwr University, Mold Rd, Wrexham LL11 2AW, Wales, UK

**Keywords:** Bioinformatics, HPC, Key-value store, Machine learning, Cloud, Metagenomics

## Abstract

We present a proof of concept implementation of the in-memory computing paradigm that we use to facilitate the analysis of metagenomic sequencing reads. In doing so we compare the performance of POSIX™file systems and key-value storage for omics data, and we show the potential for integrating high-performance computing (HPC) and cloud native technologies. We show that in-memory key-value storage offers possibilities for improved handling of omics data through more flexible and faster data processing. We envision fully containerized workflows and their deployment in portable micro-pipelines with multiple instances working concurrently with the same distributed in-memory storage. To highlight the potential usage of this technology for event driven and real-time data processing, we use a biological case study focused on the growing threat of antimicrobial resistance (AMR). We develop a workflow encompassing bioinformatics and explainable machine learning (ML) to predict life expectancy of a population based on the microbiome of its sewage while providing a description of AMR contribution to the prediction. We propose that in future, performing such analyses in ’real-time’ would allow us to assess the potential risk to the population based on changes in the AMR profile of the community.

## Highlights

• Scalable in-memory architecture for omics workflows.
• Development and evaluation of tools using in-memory key-value store.
• Using explainable machine learning on microbiome sewage data to predict life expectancy of an associated population.

## Graphical abstract

**Figure:**
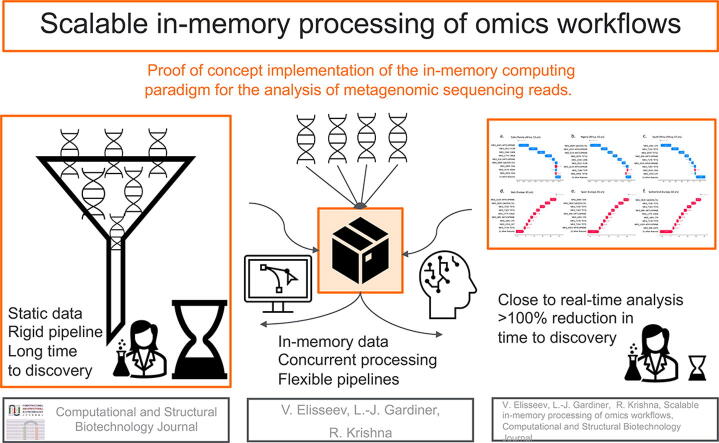

## 1 Introduction

Genomics, powered by Next Generation Sequencing (NGS) technologies, is fast emerging as an important pillar in the modern life sciences industry. Genomics is a data-intensive discipline, where each experiment can produce hundreds of gigabytes of data that needs to be computationally processed in order to derive any actionable insight. There is a substantial delay, known as *compute bottleneck*, involved in processing genomics datasets. Compute bottleneck in genomics can be due to a combination of number of factors including, vast data volume, standardized data representation, algorithmic designs, and inefficient utilization of compute resources, often available through traditional standalone, non-distributed environments. Tools are often stitched in a sequential manner, to create *workflows* that are the workhorses of the modern bioinformatics world. The bioinformatics developer community has been quick in adopting cloud-native technologies like Containers, elasticity etc. to develop *cloud-enabled* workflows that can not only run on the traditional standalone and HPC infrastructures, but can also be executed on clouds [1], [2], [3], [4].

We are gradually seeing an acceptance of cloud enabled infrastructure to meet the growing demands of genomics data. New workflows are being developed, and old ones ported in a cloud native manner to take advantage of the elastic nature of cloud, where infrastructure can be provisioned and released as per the requirement needed for an analysis. This approach is helpful in tackling the growing volume of data, as it allows us to run multiple instances of a workflow, allowing embarrassingly parallel processing of multiple datasets. However, at each workflow sub-level, there is little change in the compute bottleneck problem. Most workflows are based on the assumption that computational processing of datasets would start only after the process of data acquisition is complete. Once the entire dataset is available in a persistent manner, often on a large hard disk, it is then processed through a series of tools present in the workflow. The output from one tool is the input for the next tool in the workflow and at every instance, a secondary device based I/O is performed. This *sitting nature* of data, at each step in the workflow, poses a fundamental problem towards resolving the compute bottleneck, as not only the I/O operations can be a major time sink, but intermediate data can also be too large to move for shareability and reusability. The I/O based design also dictates the sequential nature of information flow in workflows, leading the compute to be process driven, compared to data driven. As we move towards increased adaptation of genomics in fields wider than healthcare, like pathogen surveillance, time is of the essence, and there is an acute need to accelerate the data processing problem in genomics.

Over the past few years, we have seen exciting technologies like Graphical Processing Units (GPUs), accelerators, distributed frameworks like SPARK etc. offering solutions to accelerate genomics data processing [5], [6], [7]. Much of the promise by these technologies is centred on the principle of increasing the number of compute cores and thus allowing large-scale data and compute parallelization. However, there is still the problem of massive and iterative data movement between primary and secondary storage devices and on-chip memory. Among recent cloud technologies, *in-memory databases*, based on key-value relationships, have gained traction as purpose built databases that reside in memory, compared to secondary devices. The elastic nature of cloud enables provisioning of large memory pools that can hold massive amounts of genomics data and enable minimal response time for data access and reduction in disk usage. In this study, we investigate if it is possible to utilize in-memory databases for real-life genomics data processing. We also investigate as to what are the trade-offs between using in-memory databases with respect to complementary POSIX file systems based key-value object storage technology [8]. We want to exploit the key-value representation of sequencing data to understand performance benefits, as well as possibilities of developing *event-driven* omics workflows.

In order to demonstrate the application of in-memory databases for genomics, we developed a complete bioinformatics workflow to process metagenomic sequencing reads that were derived from untreated sewage samples from sites across 60 countries (3.2 TB of uncompressed FASTQ files) to understand the key components of antimicrobial resistome in sewage with potential for indicating or even influencing lower and higher life expectancy in the population. Our analysis shows that the in-memory database driven paradigm is not only beneficial for real-time data processing, but also allows us provisions to think about alternate algorithm designs, data representation and exploitation techniques. A key advantage of using in-memory databases includes breaking the sequential nature of workflows, and allowing diverse, mutually-independent operations to be performed in parallel (e.g., functional profiling through sequence alignment could be done in parallel to k-mer based taxonomic assignment). Resultant databases can be updated with results in real-time while operations are ongoing, allowing for external tools to consume the results in real-time, as soon as they appear in the database. Architecture of this kind, allows us to envision event-driven, server-less compute paradigms that have so far, to the best of our knowledge, not been exploited in bioinformatics. We also present an extensive performance based comparison of POSIX files systems using parallel I/O with in-memory key-value storage.

## 2 In-memory paradigm in genomics

Disk storage and access incur huge latency in the processing of genomics datasets. In order to accelerate downstream data processing, the data needs to be closer to the processor and available in fast access memory devices. Historically, it was difficult to achieve this at larger scale due to cost and architectural constrains around dynamic random-access memory DRAM [9] technology. However, there have been rapid improvements in memory technologies and compute architectures that allow cost effective solutions for processing large amounts of data in significantly less time. Emergence of large, streaming data sets in various fields, coupled with rises in AI based applications, have helped large memory compute infrastructures to become mainstream. The in-memory paradigm takes advantage of the new architectural designs, where within a compute cluster, computers *pool* their RAM together to create a large virtual RAM that can be used to process a large volume of data in a much faster manner. An in-memory paradigm can minimize the slow secondary disk access and reduce latency, which is particularly important for addressing the I/O related concerns in a traditional bioinformatics workflow. The in-memory paradigm also offers an opportunity to reconsider the design of existing HPC and cloud-enabled *traditional* workflows that were designed to run on traditional low memory, secondary I/O based architectures.

**The key-value paradigm and platforms**: In-memory databases are specialized technology based on the in-memory paradigm that utilize local and distributed RAM for storing and retrieving data records. The availability of pooled RAM provides more space to hold larger volumes of data, and also reduces data access latency. Some In-memory databases work on the principles of key-value store, and are also known as key-value databases. Key-value databases are basically a form of non-relational database where data is stored in simple representation of key-value pairs. For this paper, we consider our in-memory database to be a key-value database. We take advantage of the fact that each FASTQ record, within an input FASTQ file, is an atomic unit of information, where the sequence identifier can act as key, and the sequence itself can act as value. Traditional bioinformatics tools for mapping, filtering etc produce outputs that are matched using sequence-identifiers for each input FASTQ record. Since key-value databases allow both partitioning and horizontal scaling that is not possible with relational databases, these are suitable technologies for storing intermediate data produced by different tools in a bioinformatics workflow where the output from each tool can be mapped to sequence identifiers in the input data, and be stored against the correct key in the database. As the number of tools in the workflow increase, so will the value field for each key from the input data. The in-memory database can be configured to take advantage of the elastic nature of cloud to grow as per the requirement.

**State of the art**: In-memory processing of genomics data has been attempted by various research groups using several technologies. The work by [10] presents the commonly used genomics data format, the Sequence Alignment Map (SAM) file, as ArrowSAM, an in-memory SAM format that uses the Apache Arrow framework. The authors integrate their ArrowSAM format into genome pre-processing pipelines yielding 15x and 2.4x speedups as compared to Picard and Sambamba, respectively. Gupta *et. al.* [5] proposed an accelerator, RAPID, which exploited in-memory processing solutions to enable a highly scalable, accurate and energy-efficient solution for DNA alignment. They revised a state-of-the-art alignment algorithm to make it compatible with in-memory parallel computations, and processed DNA data completely inside memory without requiring additional processing units. The authors showed that RAPID was at least 2x faster and 7x more power efficient than their benchmark accelerator BioSEAL. Castro *et. al.* [6] developed SparkBLAST, a parallelization of the sequence alignment application BLAST that employed cloud computing for provisioning of computational resources and used Apache Spark as the coordination framework. They proposed that the in-memory operations available through the Spark framework reduce the number of local I/O operations required for distributed BLAST processing resulting. ADAM [11] is another tool, providing a set of formats, APIs, and support for processing genomic data based on the combination of SPARK and cloud computing. Finally, the recent work presented by Becker *et. al.* [12] is highly relevant as the authors look beyond single steps of processing e.g., DNA sequence alignment, and demonstrate how a novel architecture, named Memory-Driven Computing (MDC), can be used for processing genomics sequencing data. They effectively have proposed ways to eliminate I/O usage from several steps of standard genomics workflows including the Samtools commands View, Sort, Markdup and Fixmate. However, this particular work uses a proprietary HPE system architecture for MDC which limits its usage for the bioinformatics community.

**Goals of this study:** It is early days for the mainstream adoption of the in-memory paradigm for genomics. As such, we designed our study to understand and evaluate the paradigm by setting the following goals:

1. to develop a proof of concept implementation of the in-memory computing paradigm using generally available components and frameworks which are available in the current technical ecosystem, as opposed to specialized hardware/software combinations. See Fig. 1 for a conceptual diagram of the proposed in-memory architecture.

**Fig. 1:**
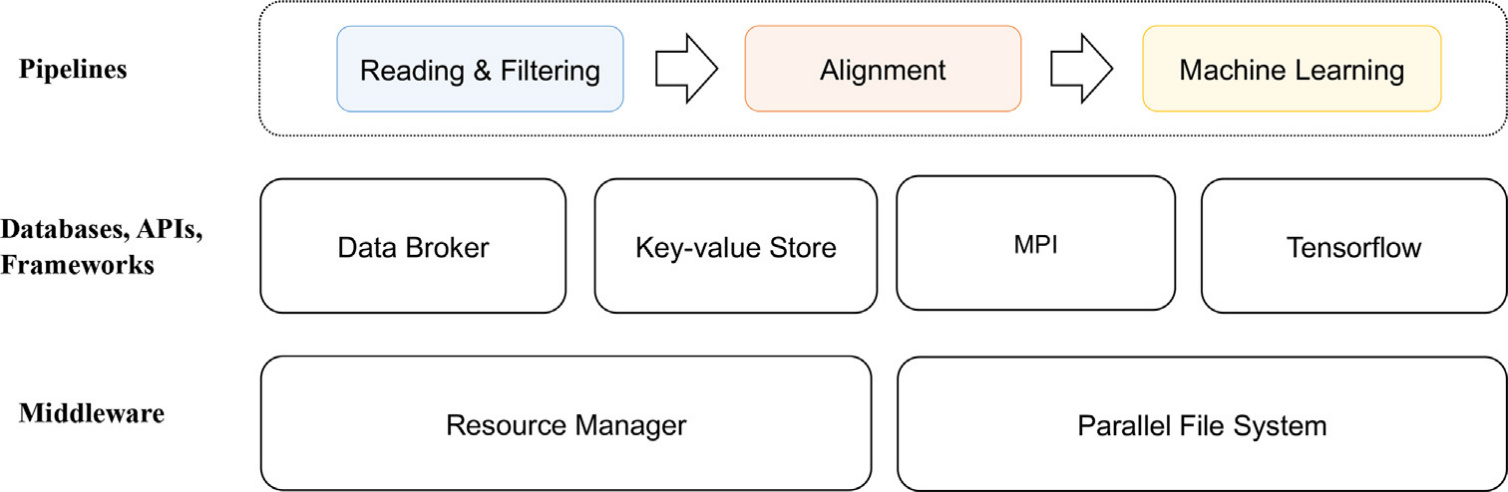
Architecture stack of the environment used to study in-memory workflows processing.
2. to highlight the benefits and opportunities for event driven and real-time data processing,
3. to show the potential for integrating existing and widely used HPC and cloud native technologies, and
4. to help evaluate in-memory technologies with respect to the traditional POSIX file interfaces.

Through our use case of processing metagenomics sewage data, we demonstrate the possibility of implementing an entire workflow using the in-memory databases and generally available compute components. We delved deeper at the key stage of duplicate removal in the workflow to show how the use of in-memory databases can enable novel and faster ways of handling the data. Importantly, we demonstrate how massive parallelization can be achieved for processing of data, tools can be run in an asynchronous manner and intermediate data can be made available as soon as it is produced; eventually paving way for data and event-driven instructions. Our demonstration makes use of traditional HPC and cloud components including an in-memory database, and implements an entire workflow, showing how it is possible to port a traditional HPC bioinformatics workflow to the proposed in-memory architecture without requiring any special arrangements. We also acknowledge that there are various flavours of in-memory databases and traditional MPI based distributed computing approaches; we have provided an extensive evaluation of comparative technologies to help understand which in-memory approach may be suitable over other in different circumstances.

## 3 Methods

### 3.1 Biological case study and the bioinformatics workflow

To showcase our proposed capability, we performed an end-to-end bioinformatic analysis of metagenomic sequencing reads using our in-memory architecture. We used this example to demonstrate how to convert a traditional bioinformatics workflow to its *in-memory counterpart* and gain scientific insights. The samples that we used as part of our biological case study included paired end metagenomic sequencing reads that were derived from untreated sewage from 79 sites across 60 countries [13]. We then identified sets of antimicrobial resistance (AMR) genes plus their coverage statistics to enable downstream Machine Learning (ML) analytics to predict key biological endpoints of interest from the microbiome of the sewage. In this case we primarily focused on the prediction of the life expectancy in years for that population. Using ML model explanation we were able to identify the key components of the antimicrobial resistome present in the sewage that are candidates for driving lower and higher life expectancy in the population. We aimed to perform the entire analytics, starting from raw data to ML prediction, using our in-memory architecture.

**Fig. 2:**
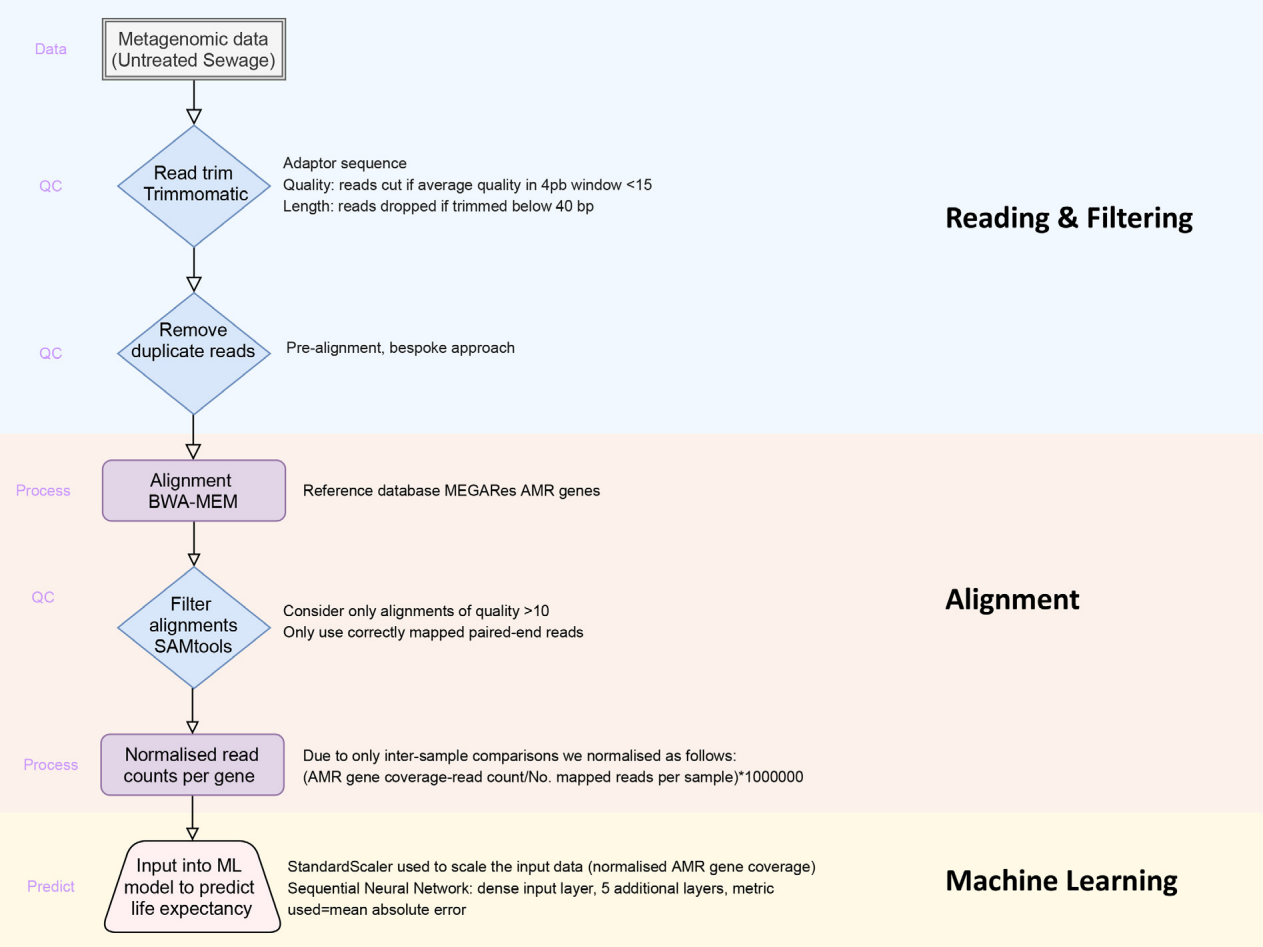
Example bioinformatic workflow to enable analysis of metagenomic samples from untreated sewage.

We developed an integrated bioinformatics and ML workflow that takes as input raw paired-end metagenomic sequencing reads and proceeds through read QC (Trimmomatic, v0.39 [14]), duplicate removal (using in-memory architecture) and read alignment (BWA-MEM, v0.7.17 [15]) against the MEGARes [16] database of 8,000 hand-curated antimicrobial resistance genes (AMRs). This read alignment is followed by alignment filtering (SAMtools, version [17]), coverage quantification (read count per AMR gene) and normalisation (to account for differing sequencing depths between samples). Fig. 2 provides a schematic of the bioinformatics workflow used for the genomics data processing and the software parameters selected where appropriate.

### 3.2 In-memory bioinformatics workflow implementation

The bioinformatics workflow was implemented using our proposed distributed in-memory architecture (Fig. 1). We have mostly used an in–house HPC cluster consisting of twenty IBM POWER^TM^compute nodes connected with 10 Gb Ethernet and 100 Gb Infiniband networks. Each node has two CPUs running at 3.69 GHz, 10 cores per CPU and 1 Tb of RAM. IBM Spectrum Scale ^TM^[18] parallel file system was used as a 50 Tb shared storage over 10 Gb network. The cluster had IBM Spectrum LSF [19] as a resource management system and OpenMPI [20] implementation of MPI. We chosen Redis ^TM^[21] for an in-memory store as one of the highest performing key-value storage solutions. We deployed Redis in cluster configuration [22] across all twenty servers with ten instances acting as masters. Such configuration allowed us to maximise number of local per node connections between Redis servers and clients, thus minimizing data movement over network.

We investigated several options of bringing in-memory storage into a workflow. We developed a number of clients using Data Broker(DBR) [23] and Hiredis [24] APIs and different Redis data types [25]. DBR provides a set of APIs, which can work with various back-ends including Redis, and as such provides a more general approach to building in-memory storage solutions. It supports both synchronous and asynchronous communications and clustering. DBR uses LIST data type for integration with Redis, which implies using of the LRANGE directive for retrieving values from a server. In contrast, Hiredis supports a wide range of Redis data types and data insertion and retrieval protocols. We also investigated applicability of HPC technologies, MPI in particular, to the in-memory processing of omics data.

**Fig. 3:**
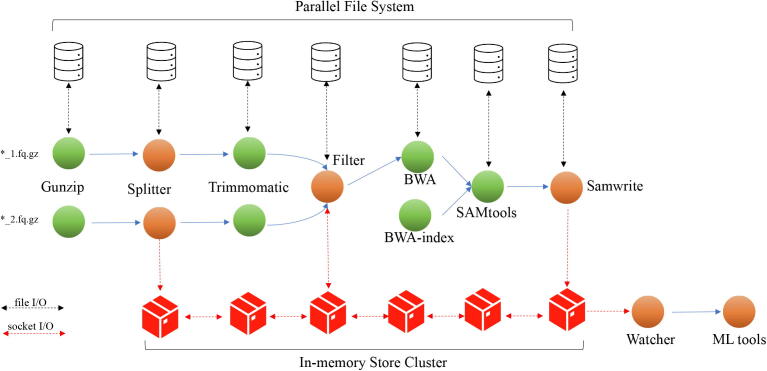
Workflow Implementation - Handling of a paired read sample in the proposed architecture. File I/O denotes reads and writes from/to a parallel file system. Socket I/O denotes reads and writes to an external in-memory key-value store.

Fig. 3 provides a schematic for the implemented *in-memory workflow*. The third-party bioinformatics tools (Green spheres) like Trimmomatic, BWA etc. were unmodified and simply used in their standard form. We developed some bespoke tools (Orange spheres) to distribute and maintain data flow in the workflow to make it compatible with the in-memory architecture. We also developed a specific example for duplicate removal using our in-memory approach to showcase how this architecture enables us to take a fresh look on an age-old problem. The bespoke tools include *Splitter*, an MPI based tool for splitting FASTQ files inspired by the algorithm proposed in [26]; *Filter*, an MPI based tool for duplicate removal, which implemented two flavours of duplicate removal algorithm using external in-memory key-value storage or local in-memory key-value based approach. *Watcher* is a tool for monitoring key-value store and data retrieval, and *ML model* a Machine-learning model for predictive analysis on microbiome (metagenomic) data. Furthermore, *Splitter* and *Filter* tools are capable of working with both file systems and external in-memory key-value storage. Overall our workflow was flexible enough to allow experimentation with different algorithms, types of storage and workflow management techniques, while being robust enough to produce tangible scientifically meaningful results.

### 3.3 Improving performance through parallelization

**Fig. 4:**
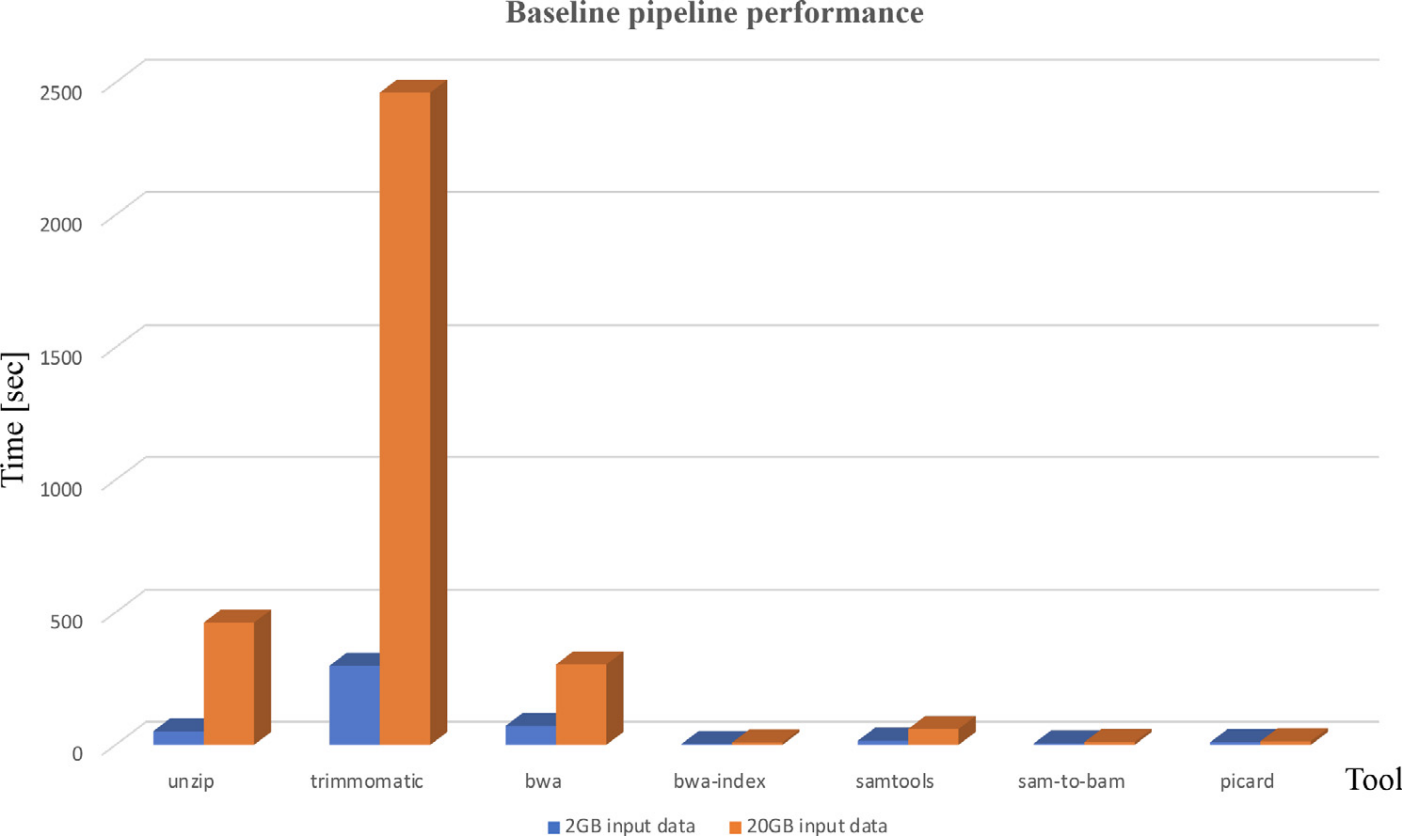
Performance profile of the base line pipeline for different input FASTQ file sizes.

Firstly, we analysed performance of the base line version of the workflow, which consists of standard tools only without our additional components in order to understand what improvements can be made without modifications to existing tools. Fig. 4 shows performance profile of the base line pipeline for 2 Gb and 20 Gb input FASTQ data sizes. It is clear that most of the workflow’s time is spent in the Trimmomatic step, hence reducing its elapsed time will provide the most benefit to improving the overall performance of the pipeline.

We observed that when running Trimmomatic, elapsed time scales linearly with the input data size (Fig. S1). Consequently, if we can split input data into multiple files and run several Trimmomatic instances concurrently, we will achieve linear speedup. We achieved splitting with our *Splitter* tool that takes a FASTQ file and divides it into several smaller FASTQ files - one per MPI rank, which can then be fed either directly to multiple instances of BWA and Samtools, or, to a *Filter* tool for additional preprocessing.

Note that *Splitter* has an option to stream FASTQ records directly to a Redis cluster instead of writing them into files. Figs. S2 and S3 show the performance of concurrent writes and reads to and from Redis server by clients using DBR or Hiredis APIs as functions of a number of MPI ranks. From these figures it can be seen that we can get linear speedup for up to 4 concurrent client–server sessions. To put this into prospective, our experimental cluster with 20 nodes, 20 cores each, can run up to 20 instances of Redis server per node processing up to 80 FASTQ files concurrently per node or 1600 FASTQ files per cluster as long as we have enough memory on each host.

### 3.4 Duplicate removal through in-memory facilities

Sequencing read duplicate removal is a critical step in a workflow, which can lead to a significant reduction in the data volume to be processed downstream and therefore an associated reduction in processing time. The earlier the duplicates can be removed the bigger gains in performance can be obtained. Therefore, we developed a duplicate removal algorithm that can be applied at any stage of the pipeline. Our in-memory architecture allowed us to develop alternate ways to perform the duplicate removal operation. Similar to [27] our algorithm explores the keys collisions property of key-value store. We experimented with two implementations of the algorithm: one using keys collision properties of Redis and another using local in-memory hash tables. Both implementations utilized MPI to take advantage of parallel processing of sequencing data. Further details of the implementation and performance results are outlined below.

#### 3.4.1 Duplicate removal using key-value store

**Fig. 5:**
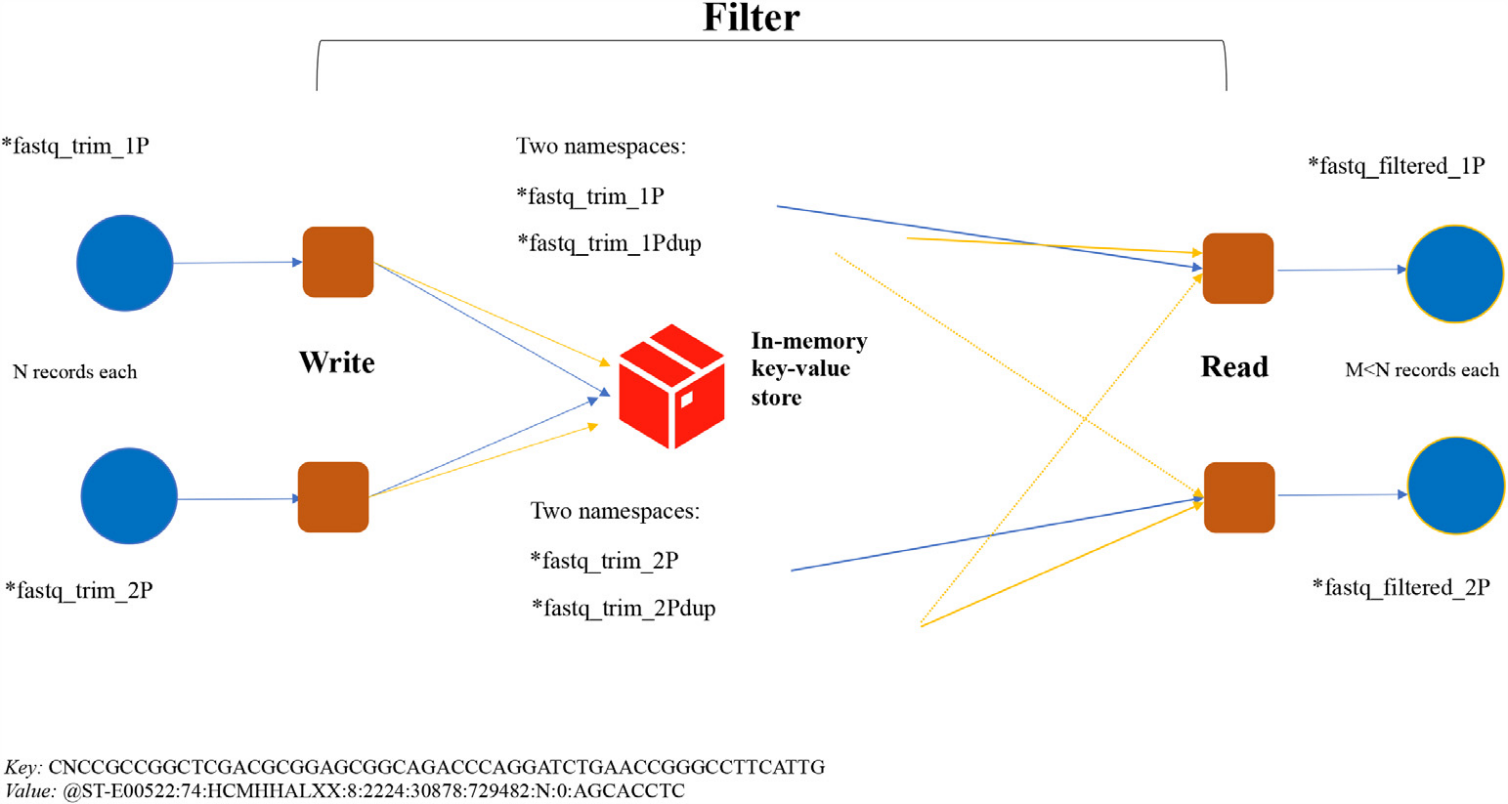
Schematic for in-memory duplicate removal using external key-value store.

Fig. 5 provides details of the external key-value storage duplicates removal algorithm, where our *Filter* tool reads data from a file system. For each pair of FASTQ files coming from Trimmomatic two instances of *Filter* are launched to write records to Redis using sequences as keys and record titles as values. What happens on the server side depends on the data type used. When LIST data type is used, each key corresponds to a unique sequence, while titles corresponding to duplicate sequences are grouped together under the same key. When SET data type is used, duplicate records are just dropped by a server. As the next step *Filter* reads records back from Redis via combination of SCAN directive to retrieve all known unique keys and either LRANGE directive for LIST data type or GET for SET data type to retrieve all or some values for each key. To make such mechanism to work as a duplicate removal filter and to keep track of duplicates, the *Filter* tool uses local in-memory algorithm to identify duplicates during the *WRITE* step and stores them in Redis under a separate namespace alongside the original data. So at the end of the *WRITE* step we have four namespaces containing data and duplicates from each input file. When reading back from Redis in a second part of the algorithm, each instance of the *Filter* tool retrieves original data from one of the data namespaces and duplicate from both namespaces from Redis and checks FASTQ records against two sets of duplicates.

**Fig. 6:**
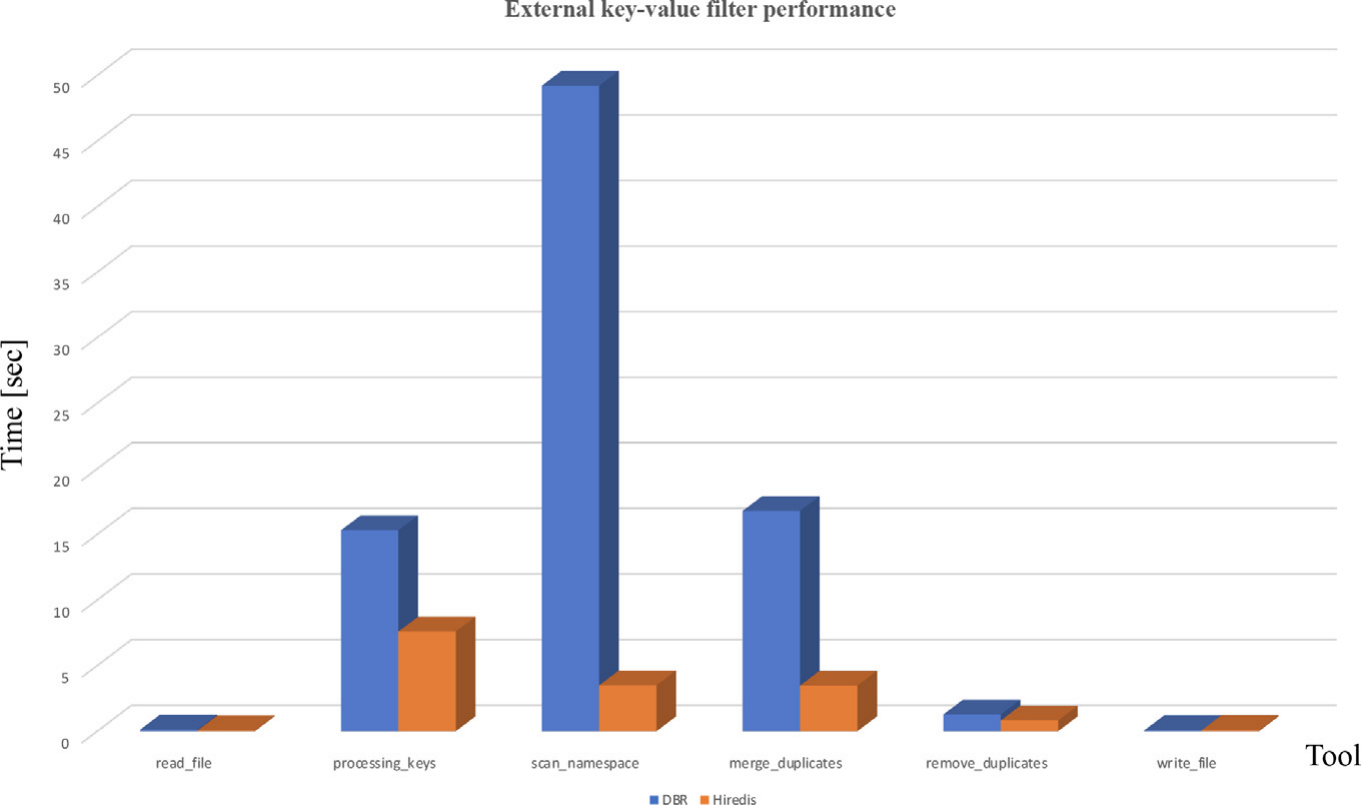
Performance profile of the external key-value store duplicate removal filter using DBR APIs (blue) and Hiredis APIs (orange). 1563892 FASTQ records, 220 bytes each have been used.

Fig. 6 shows representative performance profiles of a duplicate removal filter implemented with DBR APIs (blue) and Hiredis APIs (orange). Times are averaged across two instances of the *Filter* tool - one for each *1P/2P* input file. Description of stages of the duplicates removal is as follows:

*read*_*file* - reading of FASTQ records from a file,
*processing*_*keys* - checking for duplicates locally, sending original data and duplicates to a server,
*scan*_*namespace* - reading data from a server,
*merge*_*duplicates* - reading two sets duplicates from a server and merging them together in a single hash table,
*remove*_*duplicates* - removing duplicates,
*write*_*file* - writing resulting FASTQ records into a file.

The performance of the *Filter* is defined by the performance of the client–server communications, with Hiredis implementation offering much higher throughput compared to the DBR as we will confirm in Section 4.

Note that *Filter* throughput performance in this case can be further improved by using MPI task-based or OpenMP [28] thread-based concurrent communication between client and server components of the *Filter* tool, but this approach will also bring additional complexity in managing additional MPI tasks or OpenMP threads.

We looked at performance of communications with external key-value store, which uses TCP/UNIX sockets and compared it with performance of file based I/O. According to our experiments, writing to Redis using DBR API yielded throughput of 10 Mb/s compared to 400 Mb/s throughput on writes to a parallel file system using a single MPI task. Under the same conditions, we have obtained a throughput of 500 Mb/s for reads from a file system and 3–10 MB/s when reading (SCAN + LRANGE) from a server using DBR APIs. We have tested with 7 M keys, with each key-value pair being 220 bytes long. Our conclusion was that DBR-based implementation is too slow for dealing with massive data sets. We then looked at the Hiredis-based implementation. We experimented with the *memtier-benchmark*[29], which uses SET data type and SET + GET directives for writing and reading. *Memtier-benchmark* achieved 350 Mb/s throughput with pipelining [30] enabled. This was encouraging, so we have looked at using SCAN + LRANGE using version of the *Filter* client implemented with Hiredis APIs. We achieved approximately 200 Mb/s throughput on SCAN and 50 Mb/s throughput on LRANGE with pipelining with 7 M keys and 220 bytes long key-value pairs. This is a 10 times improvement over DBR APIs. We also tested using the same data sets and SCAN + GET directives and measured similar throughput of approximately 90 Mb/s.

#### 3.4.2 Duplicate removal using local memory

**Fig. 7:**
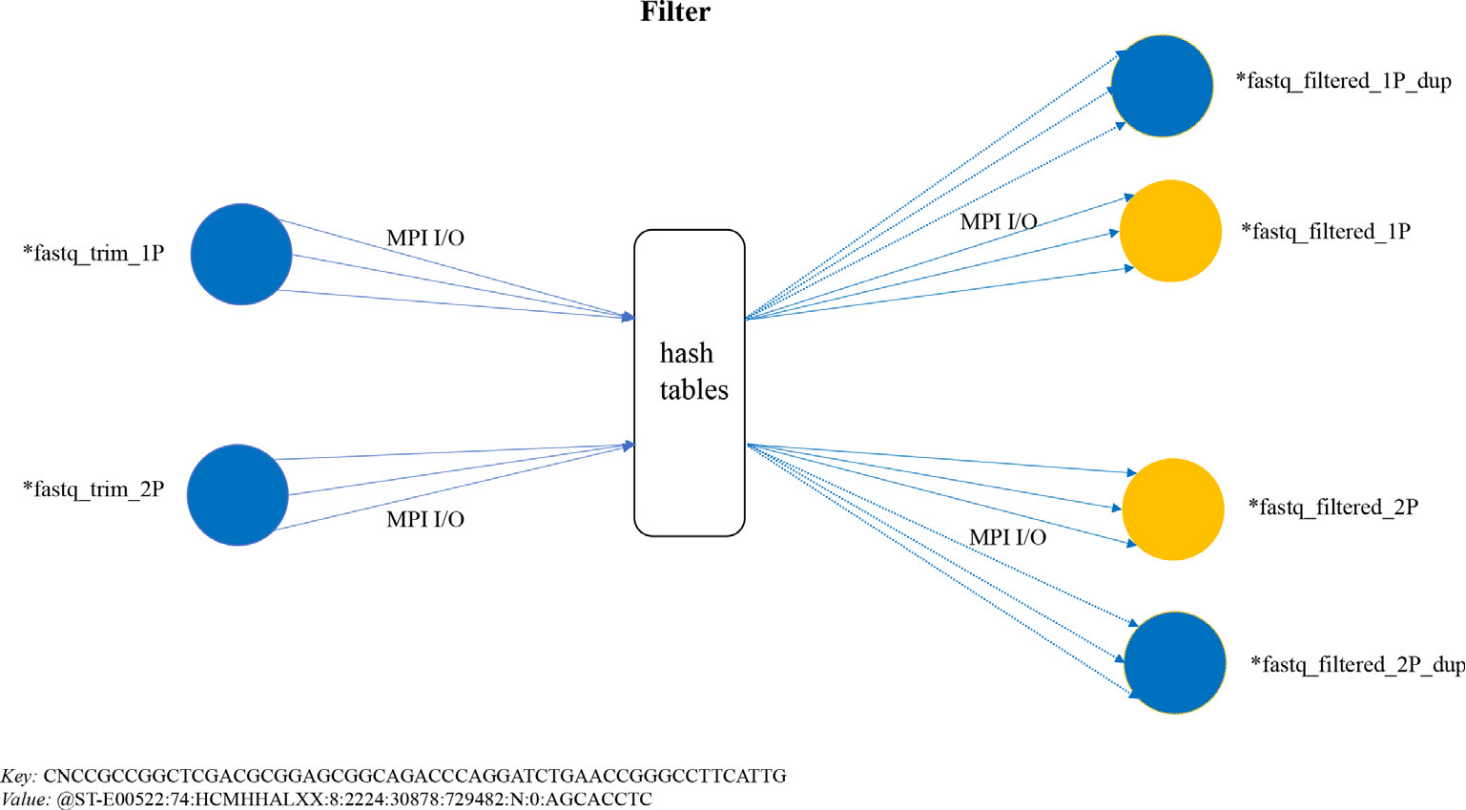
Schematic for in-memory duplicate removal using MPI and a local memory approach.

**Fig. 8:**
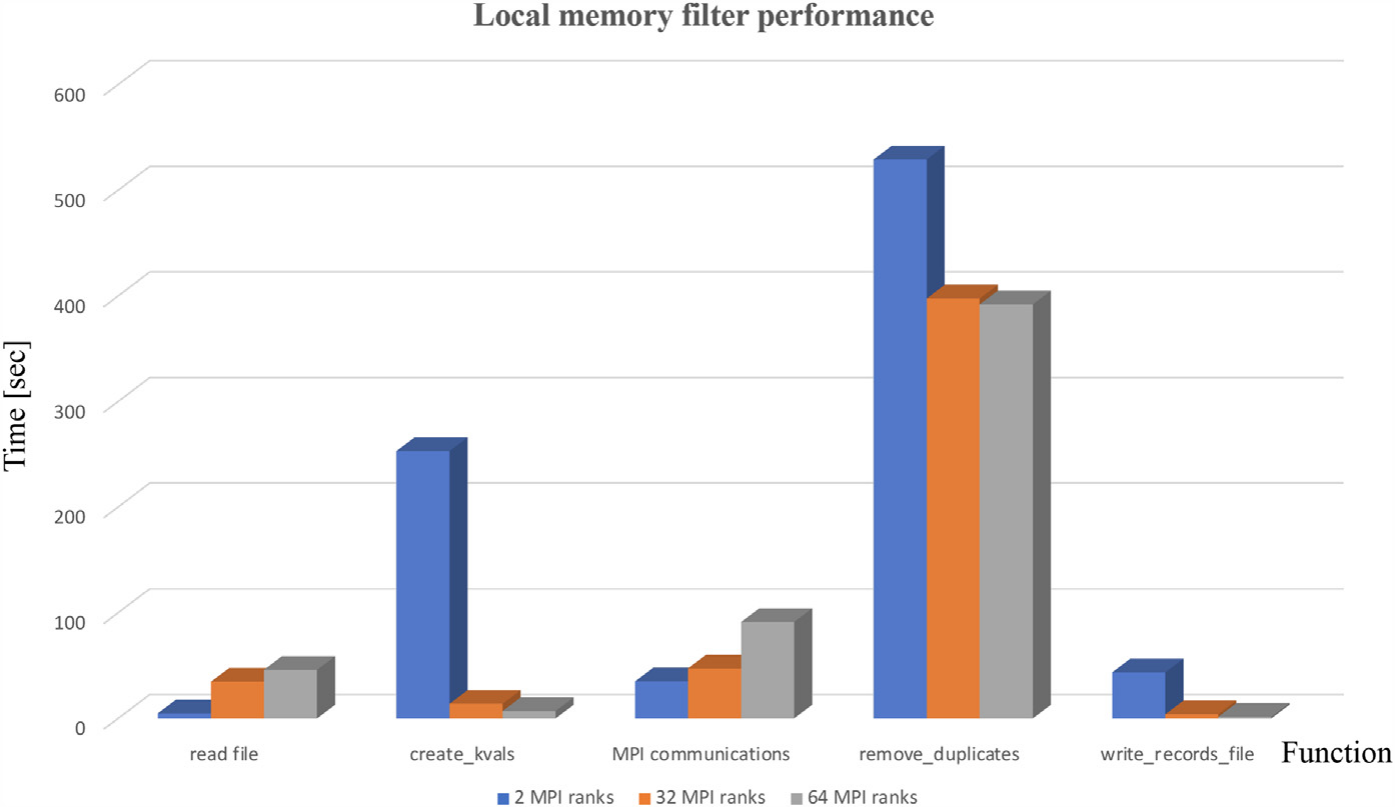
Performance profile of the local memory duplicate removal filter.

We also implemented another version of the *Filter* using only local memory for a duplicate removal algorithm, similar to the one described in [27]. Fig. 7 illustrates the main steps in the algorithm. *Filter* reads both *fastq-trim-1/2P* files created by *Trimmomatic* concurrently using MPI I/O library. Once each MPI rank has its portion of FASTQ records, it creates a local hash table and populates it using sequence line as a key. During insertion each subsequent key is checked against keys, which are already in the table and if unique, corresponding record is added to the array of unique records, if matched, a record added to the array of duplicate records. Once the process is complete, each rank has an array of unique records and an array of duplicates. Each MPI rank then broadcasts array of duplicates to all other ranks using MPI all-to-all communication. Finally each MPI ranks checks its local array of unique records against duplicates from all other ranks using the same technique as above. Once the checks are completed, final arrays of unique records from each MPI rank are written into two output files *fastq-trim-1/2P.filter* using MPI I/O. Fig. 8 shows performance profile of the MPI with local memory duplicates removal algorithm for 2, 32 and 64 MPI ranks for the same input files size (20.5 GB compressed FASTQ). Description of stages of the duplicates removal is as follows:

*read*_*file* - reading of FASTQ records from a file,
*create*_*kvals* - create key-value arrays and local duplicate arrays from input data,
*MPI communications* - exchanging duplicates among MPI tasks,
*remove*_*duplicates* - each MPI task removes all duplicates from its local FASTQ records,
*write*_*records*_*file* - writing resulting FASTQ records into a file.

We can see that the majority of the processing time is spent in actual duplicate removal and that parallelization helps to improve performance. Inter-rank communication overhead increases with the number of ranks indicating that 32 ranks is the optimum parallelization strategy in this particular use case.

### 3.5 Event-driven data processing

The in-memory database can be accessed at any stage in the workflow using the *Watcher* tool that performs LRANGE (for LIST Redis data type) or GET (for SET Redis data type) based read operations to retrieve data from given namespaces. One can trigger *Watcher* using multiple criteria, be it arrival of new data in the database, lookup for a particular key, or increase in the database size by a predetermined threshold. Since the in-memory database allows availability to all the data produced at the intermediate stages in our workflow, tools like *Watcher* can be used to collate information from various stage as soon as any tool processes a FASTQ record. For our ML case study, we used *Watcher* to retrieve data after the completion of SAMtools due to the nature of our scientific enquiry. However, for cases where real-time prediction is required through the application of online learning algorithms, *Watcher* can act as a streaming interface between the in-memory database and the ML module to facilitate immediate consumption of any intermediate data available in the database.

### 3.6 Machine Learning

Following the development of the bioinformatics workflow shown in Section 3.1, we developed, trained and tested ML models that take the normalised coverage of the AMR genes as input in order to predict the life expectancy in years for the population from which the sewage sample was derived (regression task). Firstly we developed the ML models using our full dataset that encompassed 223 individual metagenomic sequencing samples for which there was normalised sequencing coverage across a total of 7126 AMRs (features for ML). We used Scikit Learn (v3.7) [31] to build and tune the ML models. The approach used was as follows: the MinMaxScaler was used to scale the features from 0 to 1, 80% of the data was used for training and the remaining 20% was held out for testing, 5-fold cross validation was also performed on the training data using K-folds. The methods’ hyperparameters were optimized using a grid search to test a range of parameters (Table S1) for the following regressors: Logistic Regression, Random Forest, XGBoost, LightGBM, Support Vector Machine (SVM), Gaussian process, Gradient Boosting and K nearest neighbours (KNN). We then compared the different regressors to select the “best” ML model (using best parameters after fine tuning). We defined the “best” ML model for our purposes according to the lowest MAE after cross validation, balanced with the least over-fitting between training and test data.

We next used f_regression univariate linear regression tests (implemented via the Scikit Learn’s SelectKBest function) to sequentially reduce the number of AMR gene features, removing 10 each time, by choosing the most positively correlated features with the target for each subset size. Each time we reduced the feature number we re-trained and tested our “best” ML model using cross validation. This allowed us to identify a set of highly predictive subset of AMR marker genes. Finally with our defined subset of AMR marker genes we used the same approach as previously used to re-train, test and re-hyper-tune all of the ML regressors. In addition we compared the usage of the MinMaxScaler and the StandardScaler, we also compared the performance of these regressors with a neural network developed in Tensorflow with otherwise the same training and testing protocol.

## 4 Results and discussion

### 4.1 Workflow performance

This section provides statistics of processing the whole data set using our newly developed workflow. Total size of input FASTQ files was 843 GB compressed (3.2 TB uncompressed). We have used a local memory duplicate removal algorithm, so only SAM records from the final step of the pipelene were written to Redis cluster. Processing of the whole data set resulted in 42.68 GB of used Redis cluster memory with 21.34 GB of unique keys across 10 Redis masters, with 1400695 SAM records. Total elapsed time for the classical pipeline was 11.48 h. Total elapsed time for the paralellized pipeline with local memory duplicates removal was 14.7 h with 1 MPI rank and 4.9 h with 32 MPI ranks. One can see that while sequential version of our pipeline is 20% slower than a classical one due to overhead from extra steps, it outperforms the classical one by a factor of at least two with sufficient degree of parallelization. We also noted that 9.2 TB of intermediate data was generated during execution of the pipeline. Such **x15** times increase in storage requirements must be planned for and indicates a need for trade-offs between using slower, but cheaper disk storage versus faster, but more expensive in-memory storage.

### 4.2 Using ML to predict life expectancy for metagenomic sewage samples

**Fig. 9:**
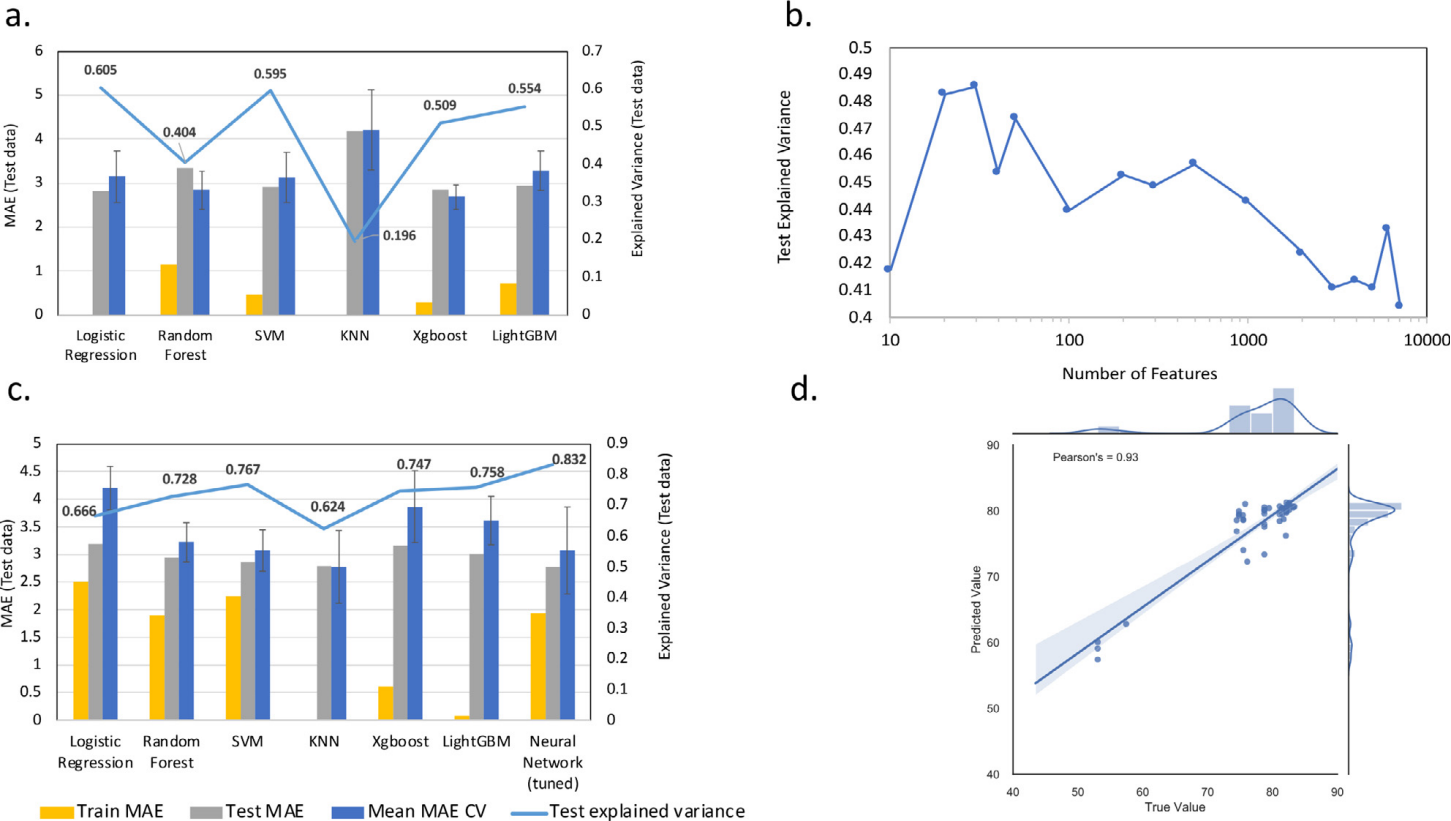
Comparison of performance of ML regression analyses. Comparing ML model performance: **(a)** using the full dataset 223 samples x 7126 AMRs, **(b)** for the best model from (a) Random Forest with sequentially reduced numbers of features, **(c)** using the 223 samples x 30 selected AMRs from (b), **(d)** showing the true versus predicted values for the test dataset when processed through the Neural Network from (c).

Our first ML analysis compared a range of regressors to predict the life expectancy in years for the population using our full dataset of 223 metagenomic sewage sequencing samples for which there was normalised sequencing coverage across a total of 7126 AMRs. The resultant best or most predictive models for each regressor (using best parameters after fine tuning) were compared based on the observed lowest Mean Absolute Error (MAE) after cross validation Fig. 9a. We observed a high degree of overfitting in our models, potentially due to the high dimensionality of our input dataset, so we prioritised the least overfitting between training and test data, which was produced using Random Forest. This we defined as our “best” model (Table S2) and it showed a MAE on the test data of 3.354, training data of 1.151 and a mean value after cross-validation of 2.841 (standard deviation 0.443), it also explained 0.404 of the variance of the test dataset (r2 0.397).

We hypothesised that reducing the number of input features into the ML (dimensionality reduction) could improve the performance of our models on the test data. As such, we sequentially reduced the number of AMR gene features by choosing the most positively correlated features with the target for each subset size (see Methods). Each time we reduced the feature number we re-trained and tested our Random Forest ML model using cross validation. Fig. 9b highlights that a reduction to only 30 AMR gene features was possible while increasing the explained variance of the test dataset to 0.486. This allowed us to identify a set of 30 highly predictive subset of AMR marker genes (Table S3). These genes fell into high-level functional MEGARes categories including “Drug and biocide resistance”, “Aminoglycosides”, “betalactams”, “Phenicol”, “Trimethoprim”, “Metronidazole”, “Sulfonamides” and “Tetracyclines”.

Finally with our defined subset of 30 AMR marker genes we used the same approach as previously used to re-train, test and re-hyper-tune all of the ML regressors plus a neural network (see Methods). The resultant best models were compared as previously Fig. 9c and the “best” model was produced using a Neural Network that comprised six layers with ReLU activation functions (see Table S2 for details). The Bayesian Optimization package from bayes_opt was used to determine the optimal layer number and neuron numbers for each layer plus their levels of dropout. The learning rate was set at 0.01, number of training epochs at 500 and the batch size at 5 to minimize loss for the validation set. This neural network showed a MAE on the test data of 2.772, training data of 1.940 and a mean value after cross-validation of 3.071 (standard deviation 0.784), it also explained 0.832 of the variance of the test dataset (r2 0.831). This amounts to a significant decrease in MAE on the test data alongside a reduction in overfitting and a marked increase in the explained variance across the test dataset. Fig. 9d highlights the high degree of correlation between true and predicted values across the test set (Pearson correlation statistic = 0.93). This trained Neural Network was then used in the last stage of our workflow in order to predict the life expectancy of new metagenomic sewage samples as they are processed by our bioinformatic workflow.

### 4.3 Using ML to gain biological insight into life expectancy

**Fig. 10:**
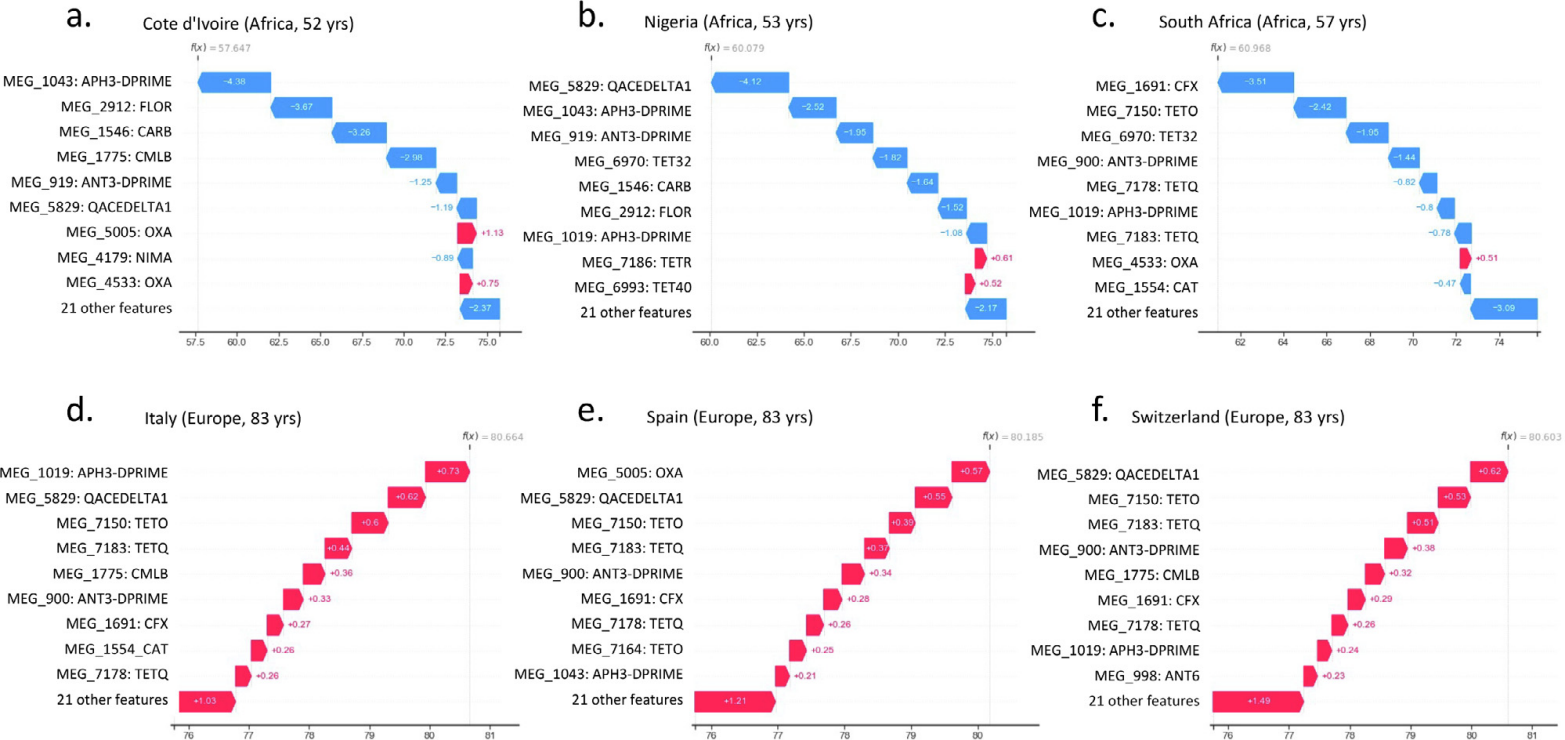
Local ML model explanation for 3 countries with the lowest life expectancy (a-c) and 3 countries with the highest life expectancy (d-f). Rows denote the AMR genes in ranked order from top to bottom according to the feature importance or impact on the models predictions. The values on the x-axis denote the SHAP calculated impact value of the related AMR gene on the models prediction of life expactancy in years for that sample.

It is known that the threat of antimicrobial resistance (AMR) is growing at an alarming rate and that the situation is perhaps aggravated in developing countries due to gross abuse of the use of antimicrobial treatments [32]. When we focus on the samples that we have in our biological case study that were derived from the 3 countries with the lowest life expectancy, they are all from African countries including Cote d’Ivoire (CIV, Africa, 52 years), Nigeria (NGA, Africa, 53 years) and South Africa (ZAF, Africa, 57 years). By comparison at the other end of the scale, focusing on the samples that were derived from the 3 countries with the highest life expectancy, these were all from European countries including Italy (ITA, Europe, 83 years), Spain (ESP, Europe, 83 years) and Switzerland (CHE, Europe, 83 years). We inspected both the accuracy of the life expectancy predictions by our ML model for each of these samples alongside the local model explanations for these predictions i.e., to ascertain which AMR genes the model prioritised in order to predict the lower or higher life expectancy’s for each of these countries (Fig. 10). These AMRs could give insight into the biological mechanism driving lower life expectancy.

All of the predictions for life expectancy for the countries were within 7 years of the real values (average within 4 years), this amounts to an average error rate of 13% for even those countries samples at the very edge (minimum and maximum) of the scale that we encountered in this study. Furthermore, in Fig. 10 when we focus on the ranked top 10 AMR genes that the model prioritised in order to predict the life expectancy for each country (ranked local explanation) we see 8 AMR genes conserved across the majority of the 3 countries with the lowest life expectancy (Fig. 10a-c) (MEG_1043: APH3-DPRIME, MEG_5829: QACEDELTA1, MEG_919: ANT3-DPRIME, MEG_2912: FLOR, MEG_1546: CARB, MEG_4533: OXA, MEG_6970: TET32, MEG_1019: APH3-DPRIME). These AMRs include beta-lactamases (CARB, OXA), aminoglycoside resistance genes that induce drug modification (APH3-DPRIME, ANT3-DPRIME), drug exporters such as drug/biocide SMR and florfenicol efflux pumps (QACEDELTA1, FLOR) and tetracycline ribosomal protection proteins (TET32). The AMRs, when seen at higher abundances, are key drivers of lower predictions of life expectancy and they span a broad range of antibiotic classes and resistance mechanisms, which could explain why various combinations of them associate with such a detrimental life expectancy phenotype. Interestingly, 3 of these AMRs (MEG_1043: APH3-DPRIME, MEG_5829: QACEDELTA1, MEG_1019: APH3-DPRIME) are seen amongst the most predictive AMRs from the 3 countries with the highest life expectancy (Fig. 10d-f). However, in this instance we noted that the presence of these AMRs has an opposite effect on the prediction i.e., they change from lowering to increasing the predicted life expectancy value respectively between the low and high life expectancy countries, this is driven by the underlying differences in AMR abundance between these groups (higher abundance generally associated with a lower life expectancy).

When we focus on the 3 countries with the highest life expectancy, we see 8 AMR genes conserved across the majority of them (Fig. 10d-f) (MEG_1019: APH3-DPRIME, MEG_5829: QACEDELTA1, MEG_7150: TETO, MEG_7183: TETQ, MEG_1775: CMLB, MEG_900: ANT3-DPRIME, MEG_1691: CFX, MEG_7178: TETQ). These AMRs are therefore likely to be key drivers of higher predictions of life expectancy, and this is driven by lower abundances of these AMR genes. Notably, all 8 of these AMRs are also seen amongst the most predictive AMRs from the 3 countries with the lowest life expectancy (Fig. 10a-c). The presence of these AMRs has an opposite effect on the prediction i.e., they change from increasing to lowering the predicted life expectancy value respectively between the high and low life expectancy countries, this is driven by the underlying differences in AMR abundance between these groups (lower abundance generally associated with a higher life expectancy).

## 5 Conclusions

We proposed design and development of a proof of concept in-memory computing paradigm for bioinformatics workflows using generally available components and frameworks including MPI and distributed in-memory key-value storage. In doing so, we found that in-memory key-value storage offers significant new opportunities for improving handling of omics by virtue of treating a sequencing record as an atomic unit of information, where results from each tool in a workflow are available immediately and concurrently. Availability of information at various ongoing stages in a workflow opens opportunities for more flexible and faster data processing.

Using our proposed architecture, we developed two variations of scalable duplicate removal algorithms, using local memory and external in-memory approaches, demonstrating how various components in the architecture can be utilized to achieve more than 100% speedups of an overall pipeline execution. We also compared performance of POSIX parallel files systems with in-memory key-value storage for omics data processing. We demonstrated that it is possible to achieve comparable performance between file I/O and socket I/O, when implementing distributed in-memory processing solutions. Our conclusions have broader implications, because key-value storage is the basis of the object storage architectures, which are widely used in cloud environments. While external in-memory solutions like Redis clusters combined with MPI based parallelization can provide a viable alternative to a traditional file systems based approach, it does increase complexity of the implementation. The latter can be alleviated to a large extent by using so-called cloud native technologies [33]. In the cloud native world Redis clusters can be easily managed as services with improved accessibility, reliability and availability. We can envision creating fully containerized workflows, using Docker [34] or other virtualization engines and deploying them in a form of micro-pipelines, where multiple instances can communicate concurrently with the distributed in-memory storage deployed on hybrid cloud-HPC architectures [35], [36], [37]. Using such building blocks we can implement extremely scalable and portable bioinformatics and cognitive analytics workflows delivered as-a-service [38].

To highlight the “real world” benefits of such an approach for event driven and real-time data processing, we presented a test biological case study that included the analysis of metagenomic sequencing reads derived from untreated sewage samples from sites across the world. Using the sewage samples, we demonstrate the potential for monitoring and gaining insight into global health threats at the population level, where we focus on the growing threat of antimicrobial resistance. Such analysis necessitated a metagenomics bioinformatics workflow for data processing combined with the downstream usage of explainable ML. We identified a small subset of key predictive AMR genes that were used to train a Neural Network to predict the life expectancy of a population based on the microbiome of its sewage, while providing a description of which AMRs contributed to this prediction and how. We propose that in future, the abundances of our defined subset of 30 AMR genes could be monitored in ’real-time’ via our bioinformatic and ML workflow as routine sequencing of the sewage microbiome is carried out. This would allow assessment of the potential risk to the population, in this case using changes in predicted life expectancy as a proxy for risk, as the AMR profile of the community changes over time. Moreover, other applications of such a workflow are possible if, for example, our ML workflow was re-trained with different genes or to predict other attributes.

## Declarations

### Availability of data and materials

The experimental datasets that were used in this study are available from the ENA Sequence Read archive study PRJEB13831.

## CRediT authorship contribution statement

**Vadim Elisseev:** Conceptualization, Methodology, Software, Resources, Visualization, Writing - original draft, Writing - review & editing. **Laura-Jayne Gardiner:** Conceptualization, Methodology, Data curation, Formal analysis, Visualization, Writing - original draft, Writing - review & editing. **Ritesh Krishna:** Conceptualization, Methodology, Writing - original draft, Writing - review & editing.

## Declaration of Competing Interest

The authors declare that they have no known competing financial interests or personal relationships that could have appeared to influence the work reported in this paper.
