# Supplementary material for: Scalable in-memory processing of omics workflows
Source: Comput Struct Biotechnol J. 2022 Apr 20;20:1914–24. doi: 10.1016/j.csbj.2022.04.014 (PMC9052061; doi:10.1016/j.csbj.2022.04.014)
Supplement: Supplementary file 1 [file mmc1.pdf]

7. Supplementary Information

7.1. Supplementary Figures

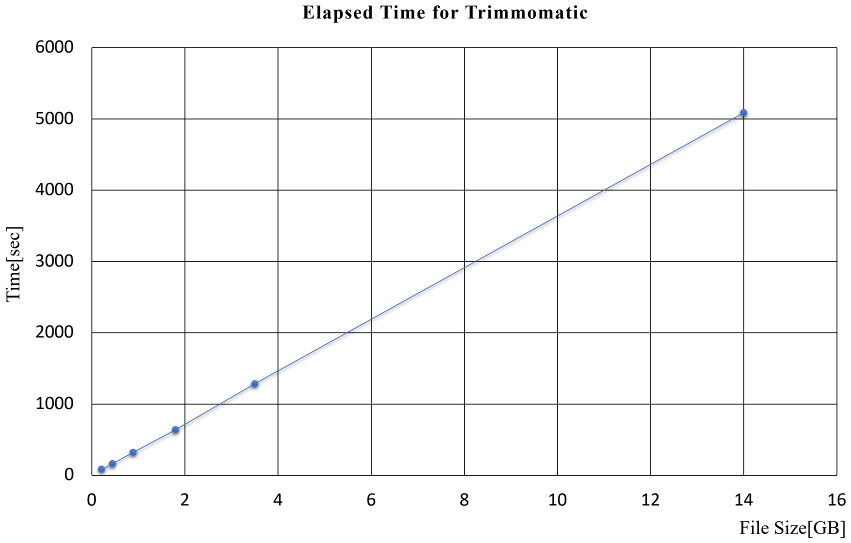

Figure S1: Elapsed time for the Trimmomatic tool as function of the input data size.

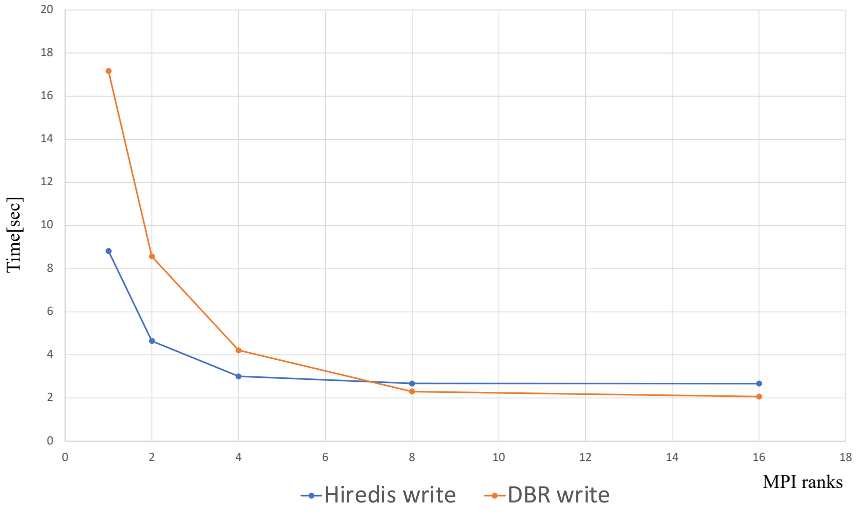

Figure S2: Concurrent writes of 852025 FASTQ records to Redis as a function of number of MPI ranks using DBR and Hiredis APIs.It is clear that increasing parallelism beyond 4 MPI ranks does not provide further gains in performance.

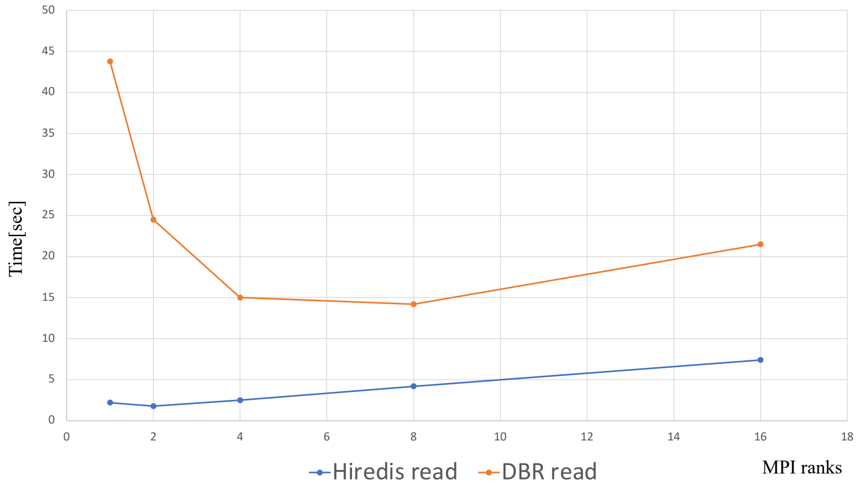

Figure S3: Concurrent reads of 852025 FASTQ records from Redis as a function of number of MPI ranks using DBR and Hiredis APIs. DBR and Hiredis demonstrate different behavior, with DBR performance improving for up to 4 MPI tasks uses, while Hiredis performance being flat for up to 4 MPI ranks. Performance degrades beyond 8 MPI tasks for both approaches, indicating that a server can no longer keep up with concurrent requests.

7.2. Supplementary Tables

Table 1: Showing the parameters that were tuned using Grid Search during hyperparameter optimization for comparison of commonly used deterministic ML regression methods.

**Regressor**	**Hyperparameter tuning**
**Logistic regression**	fit_intercept(True, False), normalize(True, False), copy_X(True, False)
**Random Forest**	criterion('mse', 'mae'), min_samples_leaf(1-10),max_depth(1-10), min_samples_split(2, 5, 10)
**SVM**	kernel('linear', 'poly', 'rbf'), degree(0-6), coef0(scipy.stats.expon(scale=1)),<br>max_iter(int(x) for x in np.linspace(start=100, stop=4000, num=10)),<br>gamma(scipy.stats.expon(scale=.1)),C(1-10)
**KNN**	n_neighbors(1-20), leaf_size(1-5),<br>weights('uniform', 'distance'), algorithm('auto', 'ball_tree','kd_tree','brute')
**XGBoost**	max_depth(2-10, 15, 20, 25), subsample(0.2, 0.5, 0.6, 0.7, 0.8, 0.9, 1),<br>learning_rate(scipy.stats.expon(scale=1)),<br>min_child_weight(scipy.stats.expon(scale=10)), max_delta_step(0, 1, 2), objective('reg:linear'),<br>gamma(scipy.stats.expon(scale=20)), n_estimators(50, 150, 200, 250, 300, 350, 400, 450, 500)
**Gradient Boosting**	criterion('mse', 'mae', 'friedman_mse'), min_samples_leaf(50, 100, 200),<br>max_depth(3, 5, 6, 7, 8, 10),<br>min_samples_split(100, 200, 300, 400, 500), max_features('sqrt'), learning_rate(0.05, 0.1, 0.2),<br>n_estimators(20, 40, 60, 80), 'clf__subsample'(0.2, 0.5, 0.6, 0.7, 0.8, 0.9, 1)
**LightGBM**	num_leaves(10, 20, 50, 100, 200), subsample(0.2, 0.5, 0.6, 0.7, 0.8, 0.9, 1),<br>min_data_in_leaf(10, 25, 50, 75, 100), max_depth(3, 5, 6, 7, 8, 10, 15, 20, 25),<br>learning_rate(scipy.stats.expon(scale=1)),<br>n_estimators(50, 150, 200, 250, 300, 350, 400, 450, 500)
**Gaussian Process**	normalize_y(True,False), copy_X_train(True, False), alpha(1e-2, 1e-4, 1e-6, 1e-8, 1e-10, 1e-12),<br>n_restarts_optimizer(10, 20, 30)

Table 2: Best performing models from ML analyses. Detailing the parameter sets used for our best performing models.

Feature Set	Regressor	Hyperparameters
**Full:**<br>**223 samples x 7126 AMRs**	Random Forest	Pipeline(memory=None, steps=[('scl', StandardScaler(copy=True, with_mean=True, with_std=True)),('clf', RandomForestRegressor(bootstrap=True, ccp_alpha=0.0, criterion='mse', max_depth=9, max_features='auto', max_leaf_nodes=None, max_samples=None, min_impurity_decrease=0.0, min_impurity_split=None, min_samples_leaf=2, min_samples_split=5, min_weight_fraction_leaf=0.0, n_estimators=100, n_jobs=None, oob_score=False, random_state=42, verbose=0, warm_start=False))], verbose=False)
**Partial:**<br>**223 samples x 30 AMRs**	Neural Network	StandardScaler(copy=True, with_mean=True, with_std=True))<br>model = Sequential()<br>model.add(Dense(88, input_dim=30, kernel_initializer='normal', activation='relu'))<br>model.add(Dropout(0.132))<br>model.add(Dense(86, activation='relu', kernel_regularizer=regularizers.l2(0.001)))<br>model.add(Dropout(0.194),)<br>model.add(Dense(48, activation='relu',kernel_regularizer=regularizers.l2(0.001)))<br>model.add(Dropout(0.247),)<br>model.add(Dense(72, activation='relu', kernel_regularizer=regularizers.l2(0.001)))<br>model.add(Dropout(0.187),)<br>model.add(Dense(84, activation='relu',kernel_regularizer=regularizers.l2(0.001)))<br>model.add(Dropout(0.218),)<br>model.add(Dense(1, kernel_initializer='normal'))<br>model.compile(loss='mean_absolute_error', optimizer='adam', metrics=[MeanAbsoluteError(name="mean_absolute_error", dtype=None)])

Table 3: 30 highly predictive AMR marker genes for ML modelling

AMR gene functional groups	Specific gene functions	MEGARes gene references
**Drug and biocide SMR efflux pumps**	QACEDELTA1	MEG_5829
**Aminoglycoside O-nucleotidyltransferases**	ANT3-DPRIME, ANT6	MEG_900, MEG_919, MEG_952 MEG_998
**Aminoglycoside O-phosphotransferases**	APH3-DPRIME	MEG_1019, MEG_1043
**Class A betalactamases**	BLAA, CARB, CFX, GES	MEG_1240, MEG_1546, MEG_1691, MEG_3131
**Chloramphenicol acetyltransferases**	CAT	MEG_1554
**Phenicol resistance MFS efflux pumps**	CMLB, FLOR	MEG_1775, MEG_2912
**Dihydrofolate reductase**	DFRF	MEG_2578
**Nim nitroimidazole reductase**	NIMA	MEG_4179
**Class D betalactamases**	NPS, OXA	MEG_4216, MEG_4533, MEG_5005
**Sulfonamide-resistant dihydropteroate synthases**	SULI	MEG_6615
**Tetracycline resistance ribosomal protection proteins**	TET32, TET40, TETO, TETQ, TETW	MEG_6970, MEG_6993, MEG_7150, MEG_7164, MEG_7178, MEG_7183, MEG_7184, MEG_7225
**Tetracycline transcriptional repressor**	TETR	MEG_7185, MEG_7186
